# Barriers to HIV Testing in Côte d'Ivoire: The Role of Individual Characteristics and Testing Modalities

**DOI:** 10.1371/journal.pone.0041353

**Published:** 2012-07-18

**Authors:** Kévin Jean, Xavier Anglaret, Raoul Moh, France Lert, Rosemary Dray-Spira

**Affiliations:** 1 Epidemiology of Occupational and Social Determinants of Health – Center for Research in Epidemiology and Population Health, INSERM U1018, Villejuif, France; 2 UMRS 1018, Université de Versailles Saint-Quentin, Villejuif, France; 3 PAC-CI Program, CHU de Treichville, Abidjan, Côte d'Ivoire; 4 INSERM U897, Université Bordeaux Segalen, Bordeaux, France; Indiana University and Moi University, United States of America

## Abstract

**Background:**

Expanding HIV testing requires a better understanding of barriers to its uptake. We investigated barriers to HIV testing in Côte d'Ivoire, taking into account test circumstances (client vs. provider-initiated).

**Methods:**

We used data from the 2005 nationally representative Demographic and Health Survey conducted in Côte d'Ivoire. Socio-demographic characteristics, sexual behaviour and knowledge and attitudes toward HIV/AIDS associated with recent (<2 years) HIV testing were identified using gender-specific univariate and multivariate logistic regressions. Among women, differential effects of barriers to testing according to test circumstance (whether they have been offered for a prenatal test or not) were assessed through interaction tests.

**Results:**

Recent HIV testing was reported by 6.1% of men and 9.5% of women (including 4.6% as part of antenatal care). Among men, having a low socioeconomic status, having a low HIV-related knowledge level and being employed [compared to those inactive: adjusted Odds Ratio (aOR) 0.46; 95% confidence interval (CI) 0.25–0.87] were associated with lower proportions of recent HIV testing. Among women without a prenatal HIV testing offer, living outside the capital (aOR 0.38; CI 0.19–0.77) and reporting a unique lifetime sexual partner constituted additional barriers to HIV testing. By contrast, among women recently offered to be tested in prenatal care, none of these variables was found to be associated with recent HIV testing.

**Conclusions:**

Various dimensions of individuals' characteristics constituted significant barriers to HIV testing in Côte d'Ivoire in 2005, with gender specificities. Such barriers are substantially reduced when testing was proposed in the framework of antenatal care. This suggests that provider-initiated testing strategies may help overcome individual barriers to HIV testing.

## Introduction

As the gatekeeper of a large panel of HIV/AIDS services, timely HIV testing plays a central role in the fight against the HIV epidemic. Among HIV-negative people, pre- and post-test counselling is an opportunity of primary prevention [1], [2]. Among HIV-positive people, it allows psychosocial support, linkage to care, treatment and prevention of the transmission, especially prevention of the mother-to-child transmission [3]–[6]. A recent study demonstrated the efficacy of early antiretroviral therapy for the prevention of HIV transmission [7], and studies based on mathematical models suggest that the *Test and Treat* strategy, consisting of treating every HIV-infected person as soon as diagnosis is made, might curb the epidemic [8]. For all these reasons, there is currently an international consensus to expand HIV testing in high-prevalence countries.

Improving HIV testing requires a better understanding of barriers and facilitators to its uptake on a voluntary basis in the general population. Indeed, although previous studies identified determinants of HIV testing in various contexts [9]–[19], some questions remain unanswered. Contextual factors, including coverage and accessibility of HIV testing facilities, play a key role [20]. Wealth and education have been consistently found to be positively associated with HIV testing across studies [9], [12], [14], [16]; however, the association with other individual characteristics including sexual behaviour [14], [18] or knowledge and attitudes toward HIV/AIDS [15], [17] has been reported more inconsistently, suggesting that these associations may vary according to epidemiological and social context as well as according to gender [15], [18]. In addition, determinants of HIV testing probably differ according to the test circumstances, either client-initiated (corresponding to the test generally referred to as Voluntary Counselling and Testing, VCT), or initiated by the health care provider either with opt-in or opt-out approaches [21]. However, most studies on determinants of HIV testing did not account for test circumstance [9], [12]–[18].

In Côte d'Ivoire, a large West African country, HIV prevalence in adults is currently estimated at 3.4% [22]. HIV testing, which relies principally on prenatal testing, VCT and diagnostic testing, has been announced free of charge by Côte d'Ivoire health authorities since 2004. However, the number of people aged 15 and older who received HIV counselling and testing in the past 12 months was estimated at 84.6 per 1000 in 2010 [22]. This is 3 times lower than that the HIV testing rate targeted by Côte d'Ivoire health authorities for 2010 (250/1000) [23], and 3 to 6 times lower than rates reported from East or South African countries (*e.g.* Ethiopia 236/1000, South Africa 240/1000, Rwanda 469/1000 [22]).

Using a nationally representative survey conducted in 2005, our objective was to identify socio-demographic characteristics, sexual behaviour and knowledge and attitudes toward HIV/AIDS that constitute barriers to HIV testing in Côte d'Ivoire, taking into account the circumstances of the test (client- or provider- initiated).

## Methods

### Study design

Data were collected in Côte d'Ivoire between August and October 2005, according to the Demographic and Health Survey (DHS) protocol [24]. To ensure national-scale representativeness, a two-stages sampling design was used and sampling weights were computed [25]. A randomized sample of household was drawn. In each randomized household, all members aged 15–49 years were proposed to participate. Those consenting were interviewed through face-to-face questionnaire. Participants were asked to provide a blood sample to assess HIV prevalence and its distribution. The results of the HIV test were not disclosed to participants, who were invited to use proximate VCT settings.

### Data collected

Participants were asked if they had ever been tested for HIV, and if so, the time since their last test. Women reporting a birth in the 2 years prior the interview were asked whether they had been offered HIV test as part of prenatal care in the past 2 years.

Socio-demographic characteristics collected at the time of interview included age, educational level (none/primary/secondary or more), family status (single/living in union/separated or widowed), employment status (student/employed/unemployed/inactive including housewives and disabled or retired individuals) and region of residence (Abidjan/other regions). Wealth was assessed through the DHS' wealth index, which takes into account a set of home-scale variables reflecting economic status (*e.g*. type of toilets, of flooring) [26]. This wealth index was classified from *Poorest* to *Richest* according to the quintiles of its distribution.

Sexual behaviour was assessed through number of lifetime sexual partners (for men: 1–3/4–10/>10; for women: 1/2/>2), age at first intercourse (<18/≥18 for men, <16/≥16 for women) and condom use at last intercourse.

The DHS questionnaire includes a set of questions on knowledge and attitudes toward HIV and people living with HIV/AIDS (PLWHA) usually used by UNAIDS [27]. A score of HIV-related knowledge was obtained by combining six questions on HIV transmission, a higher score reflecting a higher level of knowledge (See Table S1). The level of HIV-related knowledge was categorized as low, medium or high based on gender-specific tertiles of the distribution (among men: ≤3/4–5/6; among women: ≤2/3-4/5-6). Similarly, six questions regarding opinions and attitudes toward PLWHA were combined to compute a score of internalized stigma, a higher score reflecting a higher level of negative attitudes toward PLWHA (See Table S1). The level of HIV stigma was dichotomized as low (≤2) vs. high (>2).

### Statistical analysis

Analyses were restricted to participants with complete data for the variables of interest. We excluded participants reporting having never heard of AIDS, who were not asked about items related to HIV/AIDS including HIV testing, and those who had never had sexual intercourse, who were considered out of the field of the study. Because attitudes toward testing are highly susceptible to vary by gender [21], all analyses were stratified according to sex. Among women, analysis was stratified according to whether they reported having been offered an HIV test as part of antenatal care in the past two years or not. Women who had attended antenatal care in the past two years but who reported not having been proposed HIV testing as part of this care and those who did not attend antenatal care in the past two years were grouped together as a population having not been proposed for HIV test by a health provider.

Percentage of recent (<2 years) HIV testing was estimated overall and according to socio-demographic characteristics, sexual behaviour, and levels of HIV-related knowledge and stigma. Comparisons of the proportion of recent HIV testing across categories of each covariate of interest were performed using univariate and multivariate logistic regression models. Variables considered in the multivariate models included age, region of residence, and all other variables associated with recent HIV testing with a *p*-value <20% in univariate analysis.

To investigate differences according to the circumstances of testing among women, we also computed, for each covariate, a logistic model including the studied covariate, the stratification variable (*i.e.* whether the test had been proposed as part of antenatal care or not) and a term of interaction between these two variables. Significance of the interaction term was assessed through Wald Chi-Square test. All analyses were conducted using SAS statistical software version 9.1.3 (SAS Institute Inc., Cary, North Carolina, USA) and accounted for the sampling design using weights provided by DHS [25].

## Results

### Characteristics of the study population

Of a total of 5183 women and 4503 men interviewed (interview response rate: 85.9% for women, 83.6% for men), 749 women and 420 men having never heard about HIV/AIDS were excluded. An additional 347 women and 474 men having never had sexual intercourse and 205 women and 171 men with missing data were excluded. Eventually, 3882 women and 3438 men were included in the present analysis. Distributions of socio-demographic, behavioural and HIV-related characteristics are presented in Table 1. As shown in Table S2, included individuals were older, more likely to live in Abidjan, to be wealthy, highly educated, and to live in union than individuals excluded from the analysis (for each variable: *p*<0.001 among men and women).

**Table 1:** Weighted distribution of socio-demographic, behavioural and HIV-related characteristics among the included population (DHS Côte d'Ivoire, 2005).

			Men (N = 3438)	Women (N = 3882)
**Age**				
15–19			12.08	18.15
20–24			22.02	23.79
25–29			20.12	18.47
30–34			15.80	14.88
35–49			29.99	24.71
**Region**				
Abidjan			27.31	25.32
Other regions			72.69	74.68
**Wealth index**				
Poorest			15.88	15.06
Poorer			18.49	18.57
Middle			19.79	19.68
Richer			21.42	22.84
Richest			24.43	23.85
**Educational level**				
No education			32.35	51.50
Primary			24.86	28.89
Secondary			42.79	19.61
**Employment status**				
Working			74.63	65.12
Unemployed			10.54	4.96
Student			13.48	7.07
Other inactive			1.35	22.85
**Family situation**				
Single			43.42	27.00
Living in union			49.73	63.11
Separated/widowed			6.85	9.89
**Number of lifetime sexual partners**				
** Men**		**Women**		
1–3		1	33.65	32.49
4–9		2	35.82	26.19
10 or +		3 or +	30.53	41.32
**Age at first sexual intercourse**				
** Men**		**Women**		
<18		<16	55.94	47.09
18 or +		16 or +	44.06	52.91
**Reported use of condom at last intercourse**				
No/NA			72.92	86.56
Yes			27.08	13.44
**HIV stigma score**				
** Men**		**Women**		
≤2		≤2	63.44	55.20
>2		>2	36.56	44.80
**HIV-related knowledge score**				
** Men**		**Women**		
≤3		≤2	26.39	20.61
4–5		3–4	46.85	38.63
6		5–6	26.76	40.76
**Recent HIV test**				
No			93.86	90.53
Yes			6.14	9.47

Among the included population, about a fourth of the population lived in the economic capital, Abidjan. Among women (51.5%), median age was 27; it was 29 among men. Almost two thirds (63.1%) of women and half (49.7%) of men lived in union, among whom 26.0% and 14.7%, respectively, reported living in polygamous union. In median, men reported 5 lifetime sexual partners (2 among women) and becoming sexually active at 17 (16 among women). Their mean score were 4.2 for HIV-related knowledge (3.7 among women) and 2.2 for HIV stigma score (2.6 among women).

### History of HIV testing

Among men, 11.1% reported having ever been tested for HIV, and 6.1% reported a test in the past two years. Among women, these proportions were 15.1% and 9.5%, respectively. Overall, 13.0% of the women had attended prenatal care in the past two years, among whom 29.9% reported having been proposed prenatal HIV testing. The percentage of recent HIV testing was 62.7% in women who reported a testing proposal as part of prenatal care in the past two years and 5.3% in women who did not (*p*<0.001). Overall, 48.4% of women reporting a recent HIV test had been proposed HIV testing as part of prenatal care in the past two years.

Among respondents identified as HIV-positive through the HIV prevalence survey, 80.4% (75.3% of men and 82.6% of women) reported having never been tested for HIV.

### Factors associated with recent HIV testing among men

Among men, the proportions of recent HIV testing differed according to socio-demographic characteristics and levels of HIV-related knowledge and stigma (Table 2). Percentage of testing decreased with wealth (richest quintile vs. poorest: 10.4% vs. 3.2%, *p*<0.001). This percentage was also lower among men who had no or only primary education compared to those more educated, among those with a high HIV stigma score and those with a low HIV-related knowledge score. None of the sexual behaviour variables was associated with recent HIV testing in men.

**Table 2:** Characteristics associated with recent HIV testing among men in univariate and multivariate analysis (N = 3438).

	% recently tested (N = 133)	Univariate		Multivariate	
		Unadjusted OR	95% CI	Adjusted OR^1^	95% CI
**Age**					
15–19	4.96	0.47	0.19; 1.17	0.33	0.12 ; 0.93
20–24	3.24	0.30	0.14; 0.63	0.24	0.12 ; 0.49
25–29	9.97	ref.	ref.	ref.	ref.
30–34	7.55	0.74	0.33; 1.67	0.77	0.31 ; 1.95
35–49	5.43	0.52	0.26; 1.03	0.65	0.34 ; 1.21
**Region**					
Abidjan	8.5	ref.	ref.	ref.	ref.
Other regions	5.25	0.60	0.32; 1.11	1.41	0.56 ; 3.52
**Wealth index**					
* per quintile decrease*		0.72	0.59; 0.86	0.76	0.55 ; 1.03
**Educational level**					
No education	4.14	0.44	0.29; 0.67	0.82	0.47 ; 1.46
Primary	4.01	0.43	0.20; 0.93	0.68	0.28 ; 1.65
Secondary	8.89	ref.	ref.	ref.	ref.
**Employment status**					
Working	4.87	0.42	0.22; 0.79	0.56	0.25 ; 1.26
Unemployed	10.95	ref.	ref.	ref.	ref.
Student	7.90	0.70	0.29; 1.69	1.00	0.34 ; 2.94
Other inactive	20.76	2.13	0.28;15.96	2.86	0.50 ;16.53
**Family situation**					
Single	6.20	0.38	0.15; 0.96	0.39	0.11 ; 1.37
Living in union	4.88	0.29	0.15; 0.58	0.34	0.13 ; 0.87
Separated/widowed	14.84	ref.	ref.	ref.	ref.
**Number of lifetime sexual partners**					
1–3	5.83	0.81	0.35; 1.85		
4–10	5.59	0.77	0.46; 1.30		
>10	7.12	ref.	ref.		
**Age at first intercourse**					
<18	6.39	1.10	0.68; 1.79		
18 or more	5.82	ref.	ref.		
**Reported use of condom at last intercourse**					
No/NA	5.43	ref.	ref.	ref.	ref.
Yes	8.04	0.66	0.36; 1.21	0.86	0.50 ; 1.48
**HIV stigma score**					
Low	7.18	ref.	ref.	ref.	ref.
High	4.33	0.58	0.37; 0.93	0.96	0.60 ; 1.53
**HIV-related knowledge score**					
Low	3.1	0.33	0.19; 0.56	0.54	0.32 ; 0.91
Medium	6.29	0.69	0.35; 1.36	0.83	0.42 ; 1.66
High	8.87	ref.	ref.	ref.	ref.

OR Odds Ratio; CI Confidence Interval.

1 Adjusted for age, region of residence and all other variables associated with recent HIV testing with a *p*-value<20% in univariate analysis.

In multivariate analysis, age, employment status, family status and level of HIV-related knowledge were independently associated with the likelihood of recent HIV testing (Table 2). Compared to the overall group of men who were out of employment (*i.e.* those unemployed, students or inactive), men who were employed were less likely to have been tested [adjusted Odds Ratio (aOR) 0.46, 95%; Confidence Interval (CI) 0.25–0.87]. Those living in union (compared to those separated or widowed) and those with a low level of HIV-related knowledge also had a lower odds of testing. In addition, the likelihood of recent HIV testing tended to decrease with material deprivation (per wealth index quintile decrease: aOR 0.75, 95%; CI 0.56–1.03).

### Factors associated with recent HIV testing among women, according to whether they reported having been proposed HIV testing as part of antenatal care or not

As shown in Table 3, characteristics associated with recent HIV testing among women in univariate analysis differed according to whether they reported having been proposed HIV testing as part of antenatal care (N = 205) or not (N = 3677). Among women who did not report having received a prenatal testing proposal, recent testing percentage was lower among those living outside Abidjan compared to those living in the capital. Testing proportion decreased with wealth (richest quintile vs. poorest: 10.1% vs. 0.9%, *p*<0.001). This proportion was also lower among women who had no or only primary education compared to those more educated, among those with a high level of stigma and those with a low level of HIV-related knowledge. Compared to those unemployed, employed women and housewives reported lower testing percentages. Testing proportions were lower among those with a unique (vs. multiple) lifetime sexual partners (1.3% vs. 7.3%, *p*<0.001), among those who started their sexual life early and among those who did not report condom use at last intercourse.

**Table 3:** Characteristics associated with recent HIV testing among women in univariate analysis (N = 3882), according to whether they reported having been proposed an HIV test as part of antenatal care in the past two years or not.

	HIV testing proposed as part of antenatal care						Interaction *P*-value^1^
	No (N = 3677)			Yes (N = 205)			
	% recently tested (N = 115)	Unadjusted OR	95% CI	% recently tested (N = 112)	Unadjusted OR	95% CI	
**Age**							**0.1223**
15–19	3.42	0.62	0.29; 1.29	53.86	0.50	0.14 ; 1.76	
20–24	5.21	0.96	0.49; 1.87	54.48	0.51	0.20 ; 1.30	
25–29	5.43	ref.	ref.	70.17	ref.	ref.	
30–34	5.88	1.09	0.55; 2.14	83.74	2.19	0.65 ; 7.42	
35–49	6.24	1.16	0.44; 3.03	34.89	0.23	0.06 ; 0.85	
**Region**							**0.0257**
Abidjan	12.33	ref.	ref.	59.32	ref.	ref.	
Other regions	3.01	0.22	0.11; 0.44	68.37	0.68	0.26 ; 1.73	
**Wealth index**							**0.0137**
* per quintile decrease*		0.53	0.45; 0.62		0.94	0.70 ; 1.27	
**Educational level**							**0.0028**
No education	2.24	0.18	0.10; 0.30	65.01	0.81	0.36 ; 1.84	
Primary	6.60	0.55	0.34; 0.88	55.32	0.54	0.20 ; 1.46	
Secondary	11.48	ref.	ref.	69.64	ref.	ref.	
**Employment status**							**0.0240**
Working	5.28	0.35	0.12; 0.99	61.41	0.77	0.12 ; 4.92	
Unemployed	13.85	ref.	ref.	67.53	ref.	ref.	
Student	8.78	0.60	0.20; 1.79	37.65	0.29	0.03 ; 2.45	
Other inactive	2.27	0.14	0.05; 0.39	67.62	1.00	0.15 ; 6.60	
**Family situation**							**0.2682**
Single	4.93	0.90	0.34; 2.36	43.86	0.57	0.06 ; 5.62	
Living in union	5.41	0.99	0.43; 2.28	66.04	1.41	0.19;10.50	
Separated/widowed	5.45	ref.	ref.	58.02	ref.	ref.	
**Number of lifetime sexual partners**							**0.0058**
1	1.27	0.15	0.07; 0.30	66.61	1.41	0.52 ; 3.82	
2	6.04	0.74	0.37; 1.47	63.84	1.25	0.60 ; 2.60	
3 or more	7.99	ref.	ref.	58.65	ref.	ref.	
**Age at first sexual intercourse**							**0.5692**
<16	3.52	0.50	0.27; 0.90	57.85	0.68	0.32 ; 1.46	
16 or more	6.85	ref.	ref.	66.77	ref.	ref.	
**Reported use of condom at last intercourse**							**0.6322**
No/NA	4.63	0.47	0.24; 0.93	61.95	0.70	0.19 ; 2.51	
Yes	9.35	ref.	ref.	70.05	ref.	ref.	
**HIV stigma score**							**0.4194**
Low	7.40	ref.	ref.	66.22	ref.	ref.	
High	2.79	0.36	0.17; 0.94	54.48	0.61	0.27 ; 1.36	
**HIV-related knowledge score**							**0.1330**
Low	1.02	0.11	0.03; 0.34	56.42	0.70	0.23 ; 2.15	
Medium	4.07	0.44	0.22; 0.89	61.64	0.87	0.34 ; 2.24	
High	8.75	ref.	ref.	64.86	ref.	ref.	

OR Odds Ratio; CI Confidence Interval.

1 *P*-value of the interaction term of a logistic model including each covariate separately, the stratification variable and an interaction term between these two variables.

Among women who had received an HIV testing offer as part of antenatal care, no difference was observed in the percentages of recent HIV testing across the categories of the various covariates, excepted age. Terms of interaction between HIV-testing proposal and region of residence (interaction *p* = 0.0257), household economic wealth (*p* = 0.0137), educational attainment (*p* = 0.0028), employment status (*p* = 0.0240) and number of sexual partners (*p* = 0.0058) were significant, suggesting a significantly reduced associations between those variables and recent testing for women who had been offered a prenatal test compared to women who did not.

Results of the multivariate regression among women are presented in Table 4. Among women who were offered a prenatal test, none of the studied variables appeared to be independently associated with the likelihood of HIV testing. Among other women, controlling for other covariates, those living outside Abidjan were less likely to have been recently tested. Poverty, lack of education, reporting a unique (vs. multiple) lifetime sexual partners (aOR 0.30; CI 0.15–0.62) and low HIV-related knowledge level were independently associated with a decreased likelihood of recent testing.

**Table 4:** Characteristics associated with recent HIV testing among women in multivariate analysis (N = 3882), according to whether they reported having been proposed an HIV test as part of antenatal care in the past two years or not.

	HIV testing proposed as part of antenatal care			
	No (N = 3677)		Yes (N = 205)	
	Adjusted^1^ OR	[95% CI]	Adjusted^1^ OR	[95% CI]
**Age**				
15–19	0.77	0.30 ; 1.97	0.53	0.14 ; 1.94
20–24	0.85	0.43 ; 1.67	0.53	0.21 ; 1.32
25–29	ref.	ref.	ref.	ref.
30–34	0.86	0.34 ; 2.13	2.09	0.60 ; 7.21
35–49	2.01	0.73 ; 5.54	0.22	0.06 ; 0.84
**Region**				
Abidjan	ref.	ref.	ref.	ref.
Other regions	0.38	0.19 ; 0.77	0.79	0.31 ; 2.00
**Wealth index**				
* per quintile decrease*	0.83	0.69 ; 1.00		
**Educational level**				
No education	0.51	0.28 ; 0.92		
Primary	0.93	0.54 ; 1.60		
Secondary	ref.	ref.		
**Employment status**				
Working	0.49	0.21 ; 1.14		
Unemployed	ref.	ref.		
Student	0.49	0.15 ; 1.60		
Other inactive	0.30	0.11 ; 0.88		
**Number of lifetime sexual partners**				
1	0.31	0.14 ; 0.69		
2	1.06	0.52 ; 2.19		
3 or more	ref.	ref.		
**Age at first sexual intercourse**				
<16	0.69	0.37 ; 1.30		
16 or more	ref.	ref.		
**Reported use of condom at last intercourse**				
No/NA	0.81	0.37 ; 1.75		
Yes	ref.	ref.		
**HIV stigma score**				
Low	ref.	ref.		
High	0.77	0.36 ; 1.63		
**HIV-related knowledge score**				
Low	0.23	0.06 ; 0.83		
Medium	0.58	0.26 ; 1.28		
High	ref.	ref.		

OR Odds Ratio; CI Confidence Interval.

1 Adjusted for age, region of residence and all other variables associated with recent HIV testing with a *p*-value<20% in univariate analysis.

## Discussion

In this national-representative study, we observed a low HIV testing percentage overall. Barriers to HIV testing of various natures were identified, including region of residence, socioeconomic conditions (poverty, low education, employment status, stable partnership), sexual behaviour and level of HIV-related knowledge, with some gender differences. Among women, HIV testing offer as part of prenatal care was associated with reduced disparities in HIV testing across the various subgroups of the population.

We found that in 2005 in Côte d'Ivoire, recent HIV testing prevalence was 9.5% among women and 6.1% among men. These proportions appear to be low as regard to rates reported overall in low- and middle-income countries in 2007–2008 (in median: 12.3% of women and 7.2% of men tested yearly) [28]. In our study, we excluded persons having never had sexual intercourse and/or having never heard about HIV. The true percentage of testing in the whole population might be even lower. Moreover, among individuals found HIV positive in the prevalence survey, most (80.4%) reported no previous testing, underlining the need to strengthen HIV testing promotion.

As expected, proportion of recent HIV testing is much higher among women who received a prenatal testing proposal in the past two years than among those who did not (62.7% versus 5.3%, respectively). We assumed that the female population not reporting a recent prenatal testing proposal did not receive testing proposal elsewhere. To our knowledge, besides prenatal testing, no other program of provider-initiated counselling and testing were implemented in Côte d'Ivoire at the time of the study. Among women who attended prenatal care, the proportion reporting having been offered HIV testing in this context was low (29.9%), possibly resulting from a recall bias. Because characteristics associated with such a recall bias may be the same as those identified as barriers to testing, this could have led to underestimate the associations between individual characteristics and testing uptake in this group of women. However, if massive, such a recall bias would lead to a substantial overestimation of prenatal testing acceptance in our study, whereas the acceptance we observed (62.7%) was relatively low compared to those observed in other studies (79% to 95%) [29]–[31]. This suggests a limited recall bias. In Côte d'Ivoire, prenatal testing has been recommended through national guidelines since 1999; low proportion of women attending antenatal care who were offered an HIV test may be related to operational difficulties or failing in guidelines application coverage. A study conducted in 2007–2008 highlighted improvements in this coverage, with 60.3% of women attending prenatal care receiving HIV testing proposal [32].

Our study identified various kinds of factors acting as barriers to voluntary HIV testing. First, factors related to testing offer: among women, we observed an effect of the region of residence, women living in the capital, Abidjan, being more likely to have been recently tested. This could be related to the geography of the epidemic, since HIV prevalence survey showed a greater epidemic in Abidjan than elsewhere among women. Given the lack of a mapping of testing services, higher testing uptake may also reflect a higher prevention effort in the economic capital than in the rest of the country. This also appears in the proportion of pregnant women receiving a prenatal testing proposal: 44.1% in Abidjan vs. 18.4% elsewhere (*p*<0.001).

Second, socioeconomic trends in uptake of HIV testing were identified. Consistently with previous reports from other settings in sub-Saharan Africa, level of testing increased with wealth and education [9], [12], [14], [16]. These associations were particularly marked among women in our study. Several hypotheses may explain this social gradient in uptake of client-initiated testing. First, HIV prevalence has been reported to be positively correlated with socioeconomic level and education in many sub-Saharan African countries [33], [34]. In our study, the HIV prevalence was 5.2% for women with no educational attainment and 7.0% for those with a secondary or higher level (among men: 2.9% and 3.6% respectively). Thus, individuals with high education and wealth may perceive themselves more at risk, and therefore have a higher recourse to HIV testing compared to those less well-off. Second, this result might reflect the fact that testing promotion fails to reach the most deprived segments of the population [10]. Third, adverse living conditions associated with low socioeconomic level may constitute by themselves barriers to access to HIV testing. Further studies are needed to understand the process leading to social distribution of HIV testing uptake.

The lower testing level among employed men and women raises the issue of accessibility of testing services. Actually, in a previous study, impossibility to leave work was the second most frequently cited reason for never having tested [19]. An alternative hypothesis is an overrepresentation among those tested of men having interrupted job because of health reasons (and thus likely to have been tested late in the course of HIV infection). However, a sensitivity analysis conducted among men after excluding those reporting being too sick to work yielded a negative association between employment status and testing use (employed vs. not: aOR 0.53; CI 0.28–1.02), thus not supporting this hypothesis. Testing provision should meet the needs of the working population, for example by extending the opening hours of testing settings.

A poor level of HIV-related knowledge was identified as an additional predictor of not reporting recent HIV test among both men and women. A previous study conducted in Zimbabwe found a similar result among women but not men [11]; whereas another conducted in Uganda among men only found a significant relation [9]. As regard to stigma, we did find a negative association between HIV stigma and testing uptake in univariate analysis, but this association was not significant after multiadjustment. The association between stigma level and uptake of testing has been reported in other studies [15], [17]. Although it was based on validated questions for the measure of HIV-related stigma in developing countries [27], our stigma scale exhibited a low internal consistency (Cronbach's alpha = 0.51), which may explain the absence of independent association between stigma and testing uptake.

Lastly, testing trends related to sexual behaviour were identified in our study, with gender differences. An increased number of lifetime sexual partners was associated with higher likelihood of having been tested among women, but not among men. Gender-specificity of this association had been already observed [18], [19], and may reflect gendered norms related to sexual behaviour.

Women who received a prenatal testing proposal constitute a small sample (n = 205) and we may have failed to detect associations between provider-initiated testing and the studied variables because of lack of power. For instance, an *a posteriori* power calculation shows that an acceptable power (>0.8) to detect a significant association between recent testing and region of residence would have necessitated a sample of more than 470 women proposed for a prenatal test. However, even with such a small sample, the significant interactions we report between circumstances of testing and studied variables suggest a reduced effect of obstacles to HIV testing in the case of a provider-initiated testing. To our knowledge, this study is the first one to document a reduction of existing barriers to HIV testing through a provider proposal. These results were observed among pregnant women, who may have accepted the test to protect the infant. So we have to be careful in generalizing those results to testing offered in other circumstances However, studies conducted in clinical settings [35] or in general population [36] observed high acceptance rates for provider-initiated testing.

This study relies on a large nationally representative sample, with a high response rate. To our knowledge, this is the first work on the topic in Côte d'Ivoire investigating the role of such a broad range of dimensions as barriers to HIV testing. We acknowledge some limitations to this study. Data were collected in 2005 and have not been updated due to the political situation in Côte d'Ivoire. Nevertheless, UNAIDS reported limited progress in overall testing percentage (7.3% of the general population tested for 2009 only [37]). Our observations are thus likely to still be valid. In addition, DHS questionnaire lacks some information well identified in some studies to be associated with HIV-testing, *e.g.* the perception of HIV testing services [19]. Excluding participants who claimed having never heard of HIV/AIDS may have biased some of the measured associations; however, a sensitivity analysis in which they were accounted for as having not recently been tested for HIV led to similar results. Among men, the absence of association between HIV testing and sexual behaviour could be due to social desirability, since sexual behaviour are self-reported in face-to-face interviews. However, the seroprevalence survey revealed a positive association between reported number of lifetime sexual partners and HIV infection among men (HIV prevalence among those reporting 0/1 to 3/4 to 10/ more than 10 sexual partners: 0.4%/2.2%/3.3%/4.3%; Chi-Square *p*<10^−3^) which argue for an acceptable reliability of reported behaviours. Finally, the cross-sectional nature of the study impedes any causal interpretation of the measured associations.

Our findings suggest several perspectives for testing and prevention policies. HIV testing offer should be geographically expanded, particularly outside Abidjan, for instance with effective testing methods which has been experimented in recent years, such as mobile VCT [38] or home-based VCT [39]. Results of this study rely on information reported at individual-scale. Further studies are needed, especially on testing offer, in order to investigate how those results are articulated in the Ivorian testing landscape. Results on barriers to HIV testing, relative to social position, behaviour or HIV-related knowledge suggest setting up gender-specific communication campaigns, combining updated information on HIV and testing promotion. Such campaigns should make every attempt to reach disadvantaged populations, especially those with limited education and/or low income and the whole range of sexual partnerships. Last but not least, in addition to the improvement of services offering testing for pregnant women, our results support the expansion of provider-initiated testing in general. Opportunities for such a provider-initiated testing do not currently exist in Côte d'Ivoire for men, even though WHO recommends this strategy.

## Supporting Information

Table S1 **Assessment and coding rules for HIV stigma score and HIV-related knowledge score.**
(DOCX)

Table S2 **Weighted distribution of socio-demographic characteristics among the study population according to inclusion status (DHS Côte d**'**Ivoire, 2005).**
(DOCX)

## References

[1] Denison JA, O'Reilly KR, Schmid GP, Kennedy CE, Sweat MD (2008). HIV voluntary counseling and testing and behavioral risk reduction in developing countries: a meta-analysis, 1990–2005.. AIDS Behav.

[2] Cremin I, Nyamukapa C, Sherr L, Hallett TB, Chawira G (2010). Patterns of self-reported behaviour change associated with receiving voluntary counselling and testing in a longitudinal study from Manicaland, Zimbabwe.. AIDS Behav.

[3] De Cock KM, Fowler MG, Mercier E, de Vincenzi I, Saba J (2000). Prevention of mother-to-child HIV transmission in resource-poor countries: translating research into policy and practice.. JAMA.

[4] Grinstead OA, Gregorich SE, Choi KH, Coates T (2001). Positive and negative life events after counselling and testing: the Voluntary HIV-1 Counselling and Testing Efficacy Study.. AIDS.

[5] De Cock KM, Marum E, Mbori-Ngacha D (2003). A serostatus-based approach to HIV/AIDS prevention and care in Africa.. Lancet.

[6] Bendavid E, Brandeau ML, Wood R, Owens DK (2010). Comparative effectiveness of HIV testing and treatment in highly endemic regions.. Arch Intern Med.

[7] Cohen MS, Chen YQ, McCauley M, Gamble T, Hosseinipour MC (2011). Prevention of HIV-1 infection with early antiretroviral therapy.. N Engl J Med.

[8] Granich RM, Gilks CF, Dye C, De Cock KM, Williams BG (2009). Universal voluntary HIV testing with immediate antiretroviral therapy as a strategy for elimination of HIV transmission: a mathematical model.. Lancet.

[9] Gage AJ, Ali D (2005). Factors associated with self-reported HIV testing among men in Uganda.. AIDS Care.

[10] Wringe A, Isingo R, Urassa M, Maiseli G, Manyalla R (2008). Uptake of HIV voluntary counselling and testing services in rural Tanzania: implications for effective HIV prevention and equitable access to treatment.. Trop Med Int Health.

[11] Sherr L, Lopman B, Kakowa M, Dube S, Chawira G (2007). Voluntary counselling and testing: uptake, impact on sexual behaviour, and HIV incidence in a rural Zimbabwean cohort.. AIDS.

[12] Cremin I, Cauchemez S, Garnett G, Gregson S (2009). Patterns of uptake of HIV testing in Sub-Saharan Africa: 2003–2005.. A cross-country comparison of Demographic and Health Survey data Marrakech, Morocco.

[13] Hendriksen ES, Hlubinka D, Chariyalertsak S, Chingono A, Gray G (2009). Keep talking about it: HIV/AIDS-related communication and prior HIV testing in Tanzania, Zimbabwe, South Africa, and Thailand.. AIDS Behav.

[14] Peltzer K, Matseke G, Mzolo T, Majaja M (2009). Determinants of knowledge of HIV status in South Africa: results from a population-based HIV survey.. BMC Public Health.

[15] Young SD, Hlavka Z, Modiba P, Gray G, Van Rooyen H (2010). HIV-Related Stigma, Social Norms, and HIV Testing in Soweto and Vulindlela, South Africa: National Institutes of Mental Health Project Accept (HPTN 043). J Acquir Immune Defic Syndr.. http://www.ncbi.nlm.nih.gov.gate2.inist.fr/pubmed/20980913.

[16] Mitchell S, Cockcroft A, Lamothe G, Andersson N (2010). Equity in HIV testing: evidence from a cross-sectional study in ten Southern African countries.. BMC Int Health Hum Rights.

[17] Koku EF (2011). Desire for, and uptake of HIV tests by Ghanaian women: the relevance of community level stigma.. J Community Health.

[18] Venkatesh KK, Madiba P, De Bruyn G, Lurie MN, Coates TJ (2011). Who gets tested for HIV in a South African urban township? Implications for test and treat and gender-based prevention interventions.. J Acquir Immune Defic Syndr.

[19] Ostermann J, Reddy EA, Shorter MM, Muiruri C, Mtalo A (2011). Who Tests, Who Doesn't, and Why? Uptake of Mobile HIV Counseling and Testing in the Kilimanjaro Region of Tanzania.. PLoS ONE.

[20] WHO-UNAIDS (2007). Guidance on provider-initiated HIV testing and counselling in health facilities.. http://www.who.int/hiv/pub/vct/pitc2007/en/.

[21] Obermeyer CM, Osborn M (2007). The Utilization of Testing and Counseling for HIV: A Review of the Social and Behavioral Evidence.. Am J Public Health.

[22] WHO-UNAIDS-UNICEF (2011). Global HIV/AIDS Response. Epidemic update and health sector progress toward Universal Access. Progress report 2011.. http://www.who.int/hiv/pub/progress_report2011/en/index.html.

[23] Conseil National de Lutte contre le SIDA, République de Côte d'Ivoire (2011). Plan Stratégique National de Lutte contre l'infection au VIH, le SIDA et les IST 2011–2015..

[24] Institut National de la Statistique (INS) & Ministère de la Lutte contre le Sida [Côte d'Ivoire] & ORC Macro (2006). AIDS Indicators Survey, Côte d'IVoire 2005.. Calverton: INS & ORC Macro.

[25] Macro International Inc (1996). Sampling Manual.. Calverton: INS & ORC Macro.

[26] Rutstein SO, Johnson K (2004). DHS Comparative Reports. The DHS Wealth Index.. Calverton: ORC Macro.

[27] UNAIDS (2000). National AIDS Programmes: a Guide to Monitoring and Evaluation.. http://www.who.int/hiv/pub/epidemiology/en/JC427-Mon_Ev-Full_en.pdf.

[28] WHO (2010). Toward universal access: Scaling up priority HIV/AIDS intervention in health sector. Progress report 2010.. http://www.who.int/hiv/pub/2009progressreport/en/.

[29] Perez F, Zvandaziva C, Engelsmann B, Dabis F (2006). Acceptability of routine HIV testing (“opt-out”) in antenatal services in two rural districts of Zimbabwe.. J Acquir Immune Defic Syndr.

[30] Creek TL, Ntumy R, Seipone K, Smith M, Mogodi M (2007). Successful introduction of routine opt-out HIV testing in antenatal care in Botswana.. J Acquir Immune Defic Syndr.

[31] Peltzer K, Mlambo G, Phaweni K (2010). Factors determining prenatal HIV testing for prevention of mother to child transmission of HIV in Mpumalanga, South Africa.. AIDS Behav.

[32] Coffie PA, Kanhon SK, Touré H, Ettiegne-Traoré V, Stringer E (2011). Nevirapine for the Prevention of Mother-to-Child Transmission of HIV: A Nation-Wide Coverage Survey in Côte d'Ivoire.. J Acquir Immune Defic Syndr.

[33] Forston JG (2008). The Gradient in Sub-Saharan Africa: Socioeconomic Status and HIV/AIDS.. Demography.

[34] Fox AM (2010). The social determinants of HIV serostatus in sub-Saharan Africa: an inverse relationship between poverty and HIV?. Public Health Rep.

[35] Nakanjako D, Kamya M, Daniel K, Mayanja-Kizza H, Freers J (2007). Acceptance of routine testing for HIV among adult patients at the medical emergency unit at a national referral hospital in Kampala, Uganda.. AIDS Behav.

[36] Dalal S, Lee C, Farirai T, Schilsky A, Goldman T (2011). Provider-Initiated HIV Testing and Counseling: Increased Uptake in Two Public Community Health Centers in South Africa and Implications for Scale-Up.. PLoS ONE.

[37] UNAIDS (2010). Global Report: UNAIDS report on the global AIDS epidemic 2010.. http://www.unaids.org/globalreport/global_report.htm.

[38] Morin SF, Khumalo-Sakutukwa G, Charlebois ED, Routh J, Fritz K (2006). Removing barriers to knowing HIV status: same-day mobile HIV testing in Zimbabwe.. J Acquir Immune Defic Syndr.

[39] Mutale W, Michelo C, Jürgensen M, Fylkesnes K (2010). Home-based voluntary HIV counselling and testing found highly acceptable and to reduce inequalities.. BMC Public Health.

