# Supplementary material for: Barriers to HIV Testing in Côte d'Ivoire: The Role of Individual Characteristics and Testing Modalities
Source: PLoS One. 2012 Jul 18;7(7):e41353. doi: 10.1371/journal.pone.0041353 (PMC3399867; doi:10.1371/journal.pone.0041353)
Supplement: Table S1 — Assessment and coding rules for HIV stigma score and HIV-related knowledge score. (DOCX) [file pone.0041353.s001.docx]

**Table S1:** Assessment and coding rules for HIV stigma score and HIV-related knowledge score (DK=Don’t know).

| **HIV stigma score (0-6)** | | **Coding rules** | | **% answer coding for 1** |
| --- | --- | --- | --- | --- |
|  | If you knew a shopkeeper had HIV/AIDS, would you buy fresh vegetables from him/her? | No/DK=1 | Yes=0 | 52.3 |
|  | If a member of your family contracted HIV/AIDS, would you want it to remain secret? | Yes/DK=1 | No=0 | 56.7 |
|  | If a relative contracted HIV/AIDS, would you care him/her in your own home? | No/DK=1 | Yes=0 | 15.6 |
|  | If a female teacher had HIV/AIDS virus but was not ill, should she be allowed to continue teaching? | No/DK=1 | Yes=0 | 37.8 |
|  | People with HIV/AIDS should be ashamed of themselves. | Agree/DK=1 | Disagree=0 | 29.3 |
|  | People with HIV/AIDS should be blamed for bringing the disease into the community. | Agree/DK=1 | Disagree=0 | 35.9 |
|  |  |  |  |  |
|  | **Mean score (sd)= 2.4 (1.5) /6** | **Cronbach's alpha= 0.51** | |  |
|  |  |  |  |  |
|  |  |  |  |  |
| **HIV-related knowledge score (0-6)** | | **Coding rules** | | **% answer coding for 1** |
|  | Is it possible to reduce HIV infection risk by having a unique sexual partner, who is not infected and who doesn’t have other partner? | Yes=1 | No/DK=0 | 80.6 |
|  | Is it possible to contract HIV through mosquito bites? | No=1 | Yes/DK=0 | 47.9 |
|  | Is it possible to reduce HIV infection risk by using condom at each sexual intercourse? | Yes=1 | No/DK=0 | 73.8 |
|  | Is it possible to contract HIV through sharing food with an HIV-infected person? | No=1 | Yes/DK=0 | 68.4 |
|  | Is it possible to reduce HIV infection risk by abstaining from sex? | Yes=1 | No/DK=0 | 77.2 |
|  | Is it possible to contract HIV through witchcraft or supernatural means? | No=1 | Yes/DK=0 | 57.5 |
|  |  |  |  |  |
|  | **Mean score (sd)= 3.9 (1.7) /6** | **Cronbach's alpha= 0.69** | |  |
