# Supplementary material for: Barriers to HIV Testing in Côte d'Ivoire: The Role of Individual Characteristics and Testing Modalities
Source: PLoS One. 2012 Jul 18;7(7):e41353. doi: 10.1371/journal.pone.0041353 (PMC3399867; doi:10.1371/journal.pone.0041353)
Supplement: Table S2 — Weighted distribution of socio-demographic characteristics among the study population according to inclusion status (DHS Côte d'Ivoire, 2005). (DOCX) [file pone.0041353.s002.docx]

**Table S2:** Weighted distribution of socio-demographic characteristics among the study population according to inclusion status (DHS Côte d’Ivoire, 2005).

|  |  | **Men** | | | |  | **Women** | | | |
| --- | --- | --- | --- | --- | --- | --- | --- | --- | --- | --- |
|  |  | Overall population | Included | Excluded | *P value* |  | Overall population | Included | Excluded | *P value* |
|  |  | **N=4503** | **N=3438** | **N=1065** |  |  | **N=5183** | **N=3882** | **N=1301** |  |
| **Age** |  |  |  |  | *<.0001* |  |  |  |  | *<.0001* |
|  | 15-19 | 19.95 | 12.08 | 50.28 |  |  | 23.76 | 18.15 | 43.55 |  |
|  | 20-24 | 20.82 | 22.02 | 16.19 |  |  | 21.77 | 23.79 | 14.65 |  |
|  | 25-29 | 17.89 | 20.12 | 9.31 |  |  | 17.62 | 18.47 | 14.64 |  |
|  | 30-34 | 13.85 | 15.80 | 6.35 |  |  | 13.18 | 14.88 | 7.19 |  |
|  | 35-49 | 27.49 | 29.99 | 17.87 |  |  | 23.66 | 24.71 | 19.97 |  |
| **Region** |  |  |  |  | *0.005* |  |  |  |  | *0.283* |
|  | Abidjan | 26.37 | 27.31 | 22.74 |  |  | 24.97 | 25.32 | 23.76 |  |
|  | Other regions | 73.63 | 72.69 | 77.26 |  |  | 75.03 | 74.68 | 76.24 |  |
| **Wealth index** | |  |  |  | *0.0007* |  |  |  |  | *<.0001* |
|  | Poorest | 16.76 | 15.88 | 20.18 |  |  | 17.07 | 15.06 | 24.17 |  |
|  | Poorer | 18.79 | 18.49 | 19.96 |  |  | 18.30 | 18.57 | 17.33 |  |
|  | Middle | 20.08 | 19.79 | 21.21 |  |  | 18.57 | 19.68 | 14.64 |  |
|  | Richer | 20.94 | 21.42 | 19.11 |  |  | 21.23 | 22.84 | 15.55 |  |
|  | Richest | 23.42 | 24.43 | 19.54 |  |  | 24.83 | 23.85 | 28.31 |  |
| **Educational level** | |  |  |  | *<.0001* |  |  |  |  | *<.0001* |
|  | No education | 34.03 | 32.35 | 40.49 |  |  | 53.91 | 51.50 | 62.45 |  |
|  | Primary | 25.08 | 24.86 | 25.91 |  |  | 26.49 | 28.89 | 18.02 |  |
|  | Secondary | 40.89 | 42.79 | 33.60 |  |  | 19.60 | 19.61 | 19.53 |  |
| **Employment status** | |  |  |  | *<.0001* |  |  |  |  | *<.0001* |
|  | Working | 72.16 | 74.63 | 62.62 |  |  | 64.07 | 65.12 | 60.38 |  |
|  | Unemployed | 9.90 | 10.54 | 7.43 |  |  | 4.85 | 4.96 | 4.48 |  |
|  | Student | 16.49 | 13.48 | 28.12 |  |  | 8.98 | 7.07 | 15.72 |  |
|  | Other inactive | 1.45 | 1.35 | 1.83 |  |  | 22.10 | 22.85 | 19.42 |  |
| **Family situation** | |  |  |  | *<.0001* |  |  |  |  | *<.0001* |
|  | Single | 49.66 | 43.42 | 73.70 |  |  | 32.29 | 27.00 | 50.98 |  |
|  | Living in union | 44.42 | 49.73 | 23.96 |  |  | 58.99 | 63.11 | 44.44 |  |
|  | Separated/widowed | 5.92 | 6.85 | 2.34 |  |  | 8.72 | 9.89 | 4.58 |  |
